# Molecular Boolean analyses of chemokine (C–C motif) receptor 1, α_1B_-adrenoceptor and arginine vasopressin receptor 1A heteromers

**DOI:** 10.1016/j.bbrep.2025.102404

**Published:** 2025-12-05

**Authors:** Xianlong Gao, Matthias Majetschak

**Affiliations:** a Department of Surgery, University of South Florida Morsani College of Medicine, Tampa, FL, USA; b Department of Molecular Pharmacology and Physiology, University of South Florida Morsani College of Medicine, Tampa, Florida, USA

**Keywords:** Chemokine (C–C motif) receptor 1, α_1B_-adrenoceptor, Arginine vasopressin receptor 1A, GPCR heteromers, Human vascular smooth muscle cells, HEK293T cells

## Abstract

We reported previously that many chemokine receptors can form heteromeric complexes with α_1B_-adrenoceptor (α_1B_-AR) and arginine vasopressin receptor 1 A (AVPR1A). To gain initial insight into the relative proportions of receptors that may participate in the formation of such heteromers, we performed molecular Boolean (MolBoolean) analyses of receptor-receptor interactions in an expression system and in primary human aortic vascular smooth muscle cells (hVSMCs), utilizing chemokine (C–C motif) receptor 1, α_1B_-adrenoceptor and AVPR1A as representative receptor partners. In HEK293T cells co-expressing all three receptors, 60–70 % of each recombinant receptor were located proximal enough to permit heteromerization with the other two receptor partners. In primary human vascular smooth muscle cells, 30–50 % of each receptor were located proximal enough to permit heteromerization with the other two receptor partners. The MolBoolean analyses of receptor-receptor interactions provides new insights into the spatial distribution of GPCRs in the plasma membrane. Our finding that large proportions of the receptor partners may be able to participate in heteromerization supports the concept that such hetero-oligomeric complexes composed of CCR1, α_1B_-AR and AVPR1A could be of physiological relevance.

## Highlights

• MolBoolean analyses were used to assess interactions between CCR1, α_1B_-adrenoceptor and AVPR1A.
• 60–70 % of each recombinant receptor were located proximal enough to permit heteromerization with the other receptor partners in HEK293T cells.
• 30–50 % of each receptor were located proximal enough to permit heteromerization with the other receptor partners in human vascular smooth muscle cells.
• MolBoolean analyses provide insights into the spatial distribution of GPCRs.

## 1 Introduction

Evidence suggests that numerous G protein-coupled receptors (GPCRs) can form heterodimeric or hetero-oligomeric complexes in the plasma membrane, which show pharmacological behavior distinct from the individual protomers [[1], [2], [3]]. Several techniques, such as bioluminescence resonance energy transfer (BRET), proximity ligation assays (PLA), or co-immunoprecipitation assays, have been employed to assess proximity between two GPCR partners in recombinant and native systems, respectively. While these methods may indicate that some of the receptors are located within a proximity permitting direct physical interactions with a receptor partner, they do not provide information on the proportion of each receptor that is not near another receptor partner, and thus, unable to participate in heteromerization. Such information, however, is important to better understand the distribution of GPCR heteromers within the plasma membrane.

Recently, a method for the molecular Boolean (MolBoolean) analysis of protein-protein interactions has been developed and made commercially available, which fills in this gap [4]. The MolBoolean analysis enables the quantification of the proportions of two proteins that do not interact with each other, as well as the proportions of those proteins that are proximal enough to permit heteromerization [4].

We reported previously that multiple recombinantly and endogenously expressed chemokine receptors can form heteromeric complexes with α_1B_-adrenoceptor (α_1B_-AR) and arginine vasopressin receptor 1 A (AVPR1A) [[5], [6], [7], [8], [9], [10], [11], [12]]. The relative proportions of receptors that may participate in the formation of such heteromeric complexes, however, are unknown. Thus, it was the objective of this study to determine the proportions of receptors that may participate in the formation of heteromeric receptor complexes in an expression system and in primary human aortic vascular smooth muscle cells (hVSMCs) utilizing the MolBoolean method. We selected chemokine (C–C motif) receptor 1 (CCR1), α_1B_-AR and AVPR1A as representative heteromerization partners because we showed previously that these receptors can form hetero-dimeric and hetero-oligomeric complexes in recombinant systems, as assessed by BRET and bimolecular fluorescence and luminescence complementation BRET, and observed positive PLA signals for heterodimeric complexes between each of the partners in THP-1 cells, human monocytes and human vascular smooth muscle cells (hVSMCs) [9,11,12].

## 2 Materials and methods

### 2.1 Materials

Mouse anti-CCR1 (MAB145), mouse IgG (MAB004) and rabbit IgG (MAB1050) were purchased from R&D Systems (Minneapolis, MN, USA). Rabbit anti-α_1B_-AR (ab169523), rabbit anti-AVPR1A (bs-11598R) and mouse anti-AVPR1A (LS-C196728) were obtained from Abcam (Cambridge, UK), Bioss (Woburn, MA), and LSBio (Lynnwood, WA), respectively. TransIT-2020 Transfection Reagent was from Mirus Bio (Madison, WI). The GPCR plasmids encoding CCR1, α_1B_-AR and AVPR1A were as we described in detail previously [9,11,12].

### 2.2 Cell culture and transfection

HEK293T cells and human primary aortic vascular smooth muscle cells (hVSMCs, PCS-100-012) were purchased from American Type Culture Collection (ATCC). HEK293T cells were cultured in high glucose Dulbecco's modified Eagle medium supplemented with 10 % FBS, 100 U/mL penicillin, and 100 μg/mL streptomycin. hVSMCs were cultured in vascular basal media (ATCC PCS-100-030) supplemented with the vascular smooth muscle growth kit (ATCC PCS-100-042), 100 U/mL penicillin, and 100 μg/mL streptomycin. All cells were cultured in a humidified environment at 37 °C, 5 % CO_2_. HEK293T cells were seeded in a 6-well plate and co-transfected with three plasmids expressing CCR1, α_1b_-AR and AVPR1A at 0.3 μg each using TransIT-2020 Transfection Reagent. The next day, cells were replated at 10^4^ cells/well onto a 16-well chamber slide and incubated overnight before being used for the MolBoolean Assay.

### 2.3 MolBoolean assay

The assay was performed using the MolBoolean assay kit (Atlas Antibodies, Stockholm, Sweden) following the manufacturer's manual. Briefly, both hVSMCs and HEK293T cells were seeded in 16-well chamber slides. After fixation with 4 % formaldehyde and washing with phosphate buffered saline (PBS), cells were blocked with the blocking buffer from the kit. Cells were then incubated with different combinations of two primary antibodies (mouse anti-CCR1 and rabbit anti-α_1b_-AR,1:200/1:100; mouse anti-CCR1 and rabbit anti-AVPR1A, 1:200/1:100; mouse anti-AVPR1A/rabbit anti-α_1b_-AR, 1:200/1:100) at 4 °C overnight as indicated in the figure legends. As negative controls, cells were incubated as described before except that one antibody of each combination was replaced with mouse or rabbit IgG. Secondary species-specific antibodies tagged with oligonucleotides were added. Hybridization of the oligonucleotide tags to a preformed DNA circle then created double stranded DNA containing nickase recognition sites. Following a DNA nicking step, identifier oligonucleotides were incorporated into the DNA circle, which then contains information on whether both or only one receptor interacted with the DNA circle. After rolling circle amplification, which creates concatemeric products (rolling circle amplification products, RCPs), two distinct fluorophore-labeled reporter oligonucleotides were hybridized. Slides were then mounted in DuoLink mounting medium with DAPI. Images were captured with a Keyence microscope (BZ-X710) on three channels (blue, red and far red) with a 60x/1.5 oil objective at high resolution. The wavelengths were blue - λ_excitation/emission (em/ex)_ 360 ± 20/460 ± 25 nm, red - λ_em/ex_ 560 ± 20/630 ± 37 nm and far red - λ_em/ex_ 620 ± 30/700 ± 37 nm. The far-red channel is presented as a pseudo-color green.

The red RCPs represent signals from mouse antibodies and green RCPs represent signals from rabbit antibodies. At least 10 non-overlapping images were randomly acquired for each well in every experiment and the same settings were performed for the same experiment. To analyze the numbers of RCPs, images were first deconvoluted with ImageJ with Parallel Spectral Deconvolution v1.12 plugin and then analyzed and counted with CellProfiler with a pipeline developed by Atlas Antibodies. Schematic representations of the MolBoolean principle for detection of interacting and free proteins have been published [4,13] and are available at the manufacturer's website.

### 2.4 Data analysis and statistics

RCPs in each channel (red, green or overlapping yellow RCPs) were analyzed as total numbers or percentage of RCPs of the sum of RCPs in all channels (% of total). RCPs were graphed as scatter dot plots with mean and standard deviation indicated by horizontal lines. RCP signals per cell were calculated as the number of RCPs per nucleus in each vision field. Data are described as mean with standard deviation. All data analyses were performed with the GraphPad Prism 10 for macOS software, Version 10.6.0, August 12, 2025. The mean numbers or percentages of RCPs from each individual experiment were analyzed utilizing unpaired *t*-test with Welch's correction or one-way ANOVA with Dunnett's multiple comparisons test. A 2-tailed p < 0.05 was considered significant.

## 3 Results

We first analyzed the number of RCP signals per cell for each individual receptor when MolBoolean staining was performed with combinations of rabbit and mouse antibodies for each receptor-receptor arrangement. While all rabbit antibodies were used in combination with mouse anti-CCR1, all mouse antibodies were employed in combination with rabbit anti-α_1B_-AR. Parallel experiments in which one of the anti-GPCR antibodies was replaced with rabbit or mouse IgG served as negative controls.

**Fig. 1:**
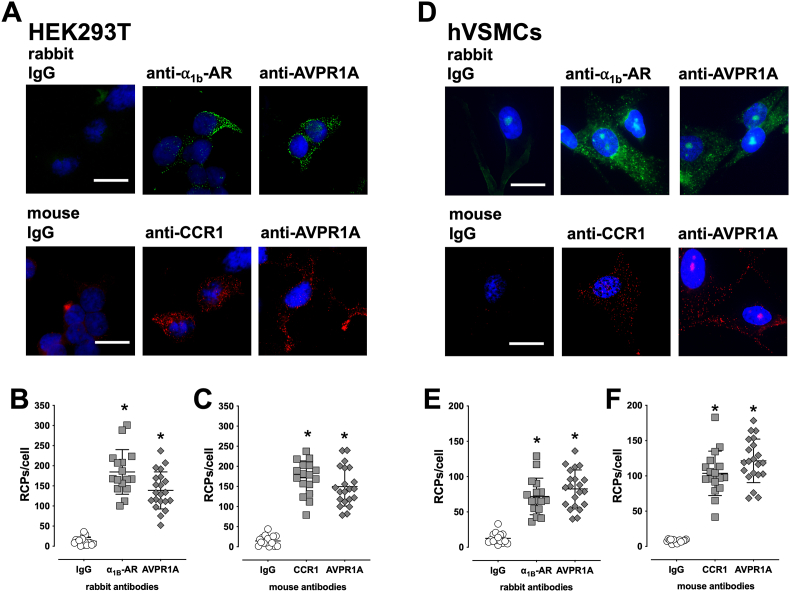
Quantification of rolling circle amplification products (RCPs) after MolBoolean staining of recombinant and endogenously expressed CCR1, α_1B_-AR and AVPR1A. **A/D.** HEK293T cells co-transfected with CCR1, α_1B_-AR and AVPR1A (**A**) or hVSMCs (**D**) were used for MolBoolean staining. Top: Cells were stained with rabbit IgG, rabbit anti-α_1B_-AR or rabbit anti-AVPR1A in combination with mouse anti-CCR1. Bottom: Cells were stained with mouse IgG, mouse anti-CCR1 or mouse anti-AVPR1A in combination with rabbit anti-anti-α_1B_-AR. Images show merged channels (blue and pseudo-color green or blue and red) and are representative of three independent experiments performed on different days. **B/C**. Quantification of RCPs/cell after MolBoolean staining of HEK293T cells, as in A, with rabbit (**B**) or mouse (**C**) antibodies. N = 16–21 vision fields from n = 3 independent experiments were analyzed per antibody. *: p < 0.05 vs. rabbit or mouse IgG. **E/F**. Quantification of RCPs/cell after MolBoolean staining of hVSMCs, as in D, with rabbit (**E**) or mouse (**F**) antibodies. N = 15–21 vision fields from n = 3 independent experiments were analyzed per antibody. *: p < 0.05 vs. rabbit or mouse IgG. Scale bars: 20 μm.

Fig. 1A shows representative images of HEK293T cells that were co-transfected with all three receptors, and Fig. 1B/C show the quantification of RCP signals from three independent experiments when cells were labeled with rabbit (Fig. 1B) or mouse (Fig. 1C) antibodies. While the number RCPs/cell after staining of cells with rabbit IgG averaged 12.4 ± 9.9 RCPs/cell, staining with rabbit anti-α_1B_-AR and rabbit anti-AVPR1A resulted in 184.6 ± 55 RCPs/cell and 138.8 ± 46 RCPs/cell, respectively (p < 0.05 vs. staining with rabbit IgG for both). After staining of cells with mouse IgG, we observed 15 ± 5 RCPs/cell, whereas 171.6 ± 41.9 and 149.6 ± 48.2 RCPs/cell were detectable after staining with mouse anti-CCR1 and mouse anti-AVPR1A, respectively (p < 0.05 vs. staining with mouse IgG for both).

Fig. 1D shows representative images of hVSMCs after MolBoolean staining with rabbit and mouse antibodies, and Fig. 1E/F show the quantification of RCP signals per cell from three independent experiments. Similar to our observations in HEK293T cells, we detected comparable numbers of RCPs/cell after MolBoolean staining of hVSMCs with rabbit IgG (12.5 ± 7.8 RCPs/cell) or mouse IgG (7.6 ± 2.2). When hVSMCs were labeled with rabbit anti-α_1B_-AR, we detected 71.9 ± 25.8 RCPs/cell, and 82.5 ± 26.8 RCPs/cell, 103.7 ± 31.4 RCPs/cell and 121.3 ± 30.8 RCPs/cell after labeling cells with rabbit anti-AVPR1A, mouse anti-CCR1 or mouse anti AVPR1A, respectively (p < 0.05 vs. rabbit or mouse IgG for all).

**Fig. 2:**
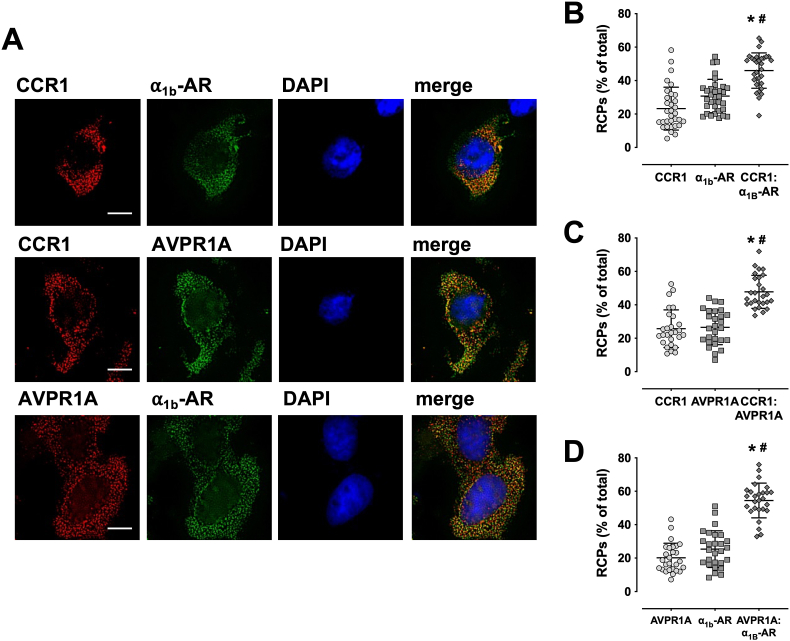
Molecular Boolean analyses of recombinantly expressed CCR1, α_1B_-AR and AVPR1A. **A**. HEK293T cells were co-transfected with CCR1, α_1B_-AR and AVPR1A and used for MolBoolean staining, as described. Images are representative of three independent experiments performed on different days. Cells were imaged (from left to right) on red, far-red (shown as pseudo-color green) and blue channels (DAPI nuclear counterstain). Merge: overlay of images from 3 channels, overlapping RCPs are displayed in yellow. Scale bars: 10 μm. **B.-D.** Quantification of RCPs from n = 3 independent experiments per condition, as in A. Horizontal lines indicate mean and SEM. RCPs were quantified as % of all (red, green and yellow) RCPs. N = 30 vision fields from n = 3 independent experiments were analyzed per condition. **B.** Cells stained for CCR1 (red RCPs) and α_1B_-AR (green RCPs). CCR1:α_1B_-AR: overlapping RCPs (yellow). *: p < 0.05 vs. CCR1. #:p < 0.05 vs. α_1B_-AR. **C.** Cells stained for CCR1 (red RCPs) and AVPR1A (green RCPs). CCR1:AVPR1A: overlapping RCPs (yellow). *: p < 0.05 vs. CCR1. #:p < 0.05 vs. AVPR1A. **D.** Cells stained for AVPR1A (red RCPs) and α_1B_-AR (green RCPs). AVPR1A:α_1B_-AR: overlapping RCPs (yellow). *: p < 0.05 vs. AVPR1A, #:p < 0.05 vs. α_1B_-AR.

Fig. 2A shows representative images of the individual fluorescent channels of HEK293T cells co-transfected with the three receptor partners and Fig. 2B–D the quantification of the RCPs from three independent MolBoolean experiments per condition. In cells stained with anti-CCR1 and anti-α_1B_-AR antibodies (Figs. 2B), 23.3 ± 12.8 % of RCPs represented CCR1 alone, 30.7 ± 9.9 % of RCPs represented α_1B_-AR alone and 46 ± 10.5 % of RCPs represented CCR1 and α_1B_-AR that are expressed in a proximity permitting formation of heteromeric receptor complexes (= overlapping RCPs, yellow). In HEK293T cells stained with anti-CCR1 and anti-AVPR1A antibodies (Fig. 1C), overlapping RCPs represented 47.8 ± 9.8 % of all RCPs, whereas 25 ± 11 % of RCPs and 26.5 ± 10.4 % of RCPs represented CCR1 and AVPR1A alone, respectively. In cells stained with anti-AVPR1A and anti-α_1B_-AR antibodies (Figs. 1D), 54.4 ± 10.4 % of all RCPs were detectable as overlapping RCPs, whereas 20.7 ± 8.7 % and 25.3 ± 11 of RPCs represented AVPR1A and α_1B_-AR in non-overlapping RCPs, respectively. For each antibody combination, the overlapping RCP signals in HEK29T cells were significantly higher than the RCP signals representing non-interacting receptors.

**Fig. 3:**
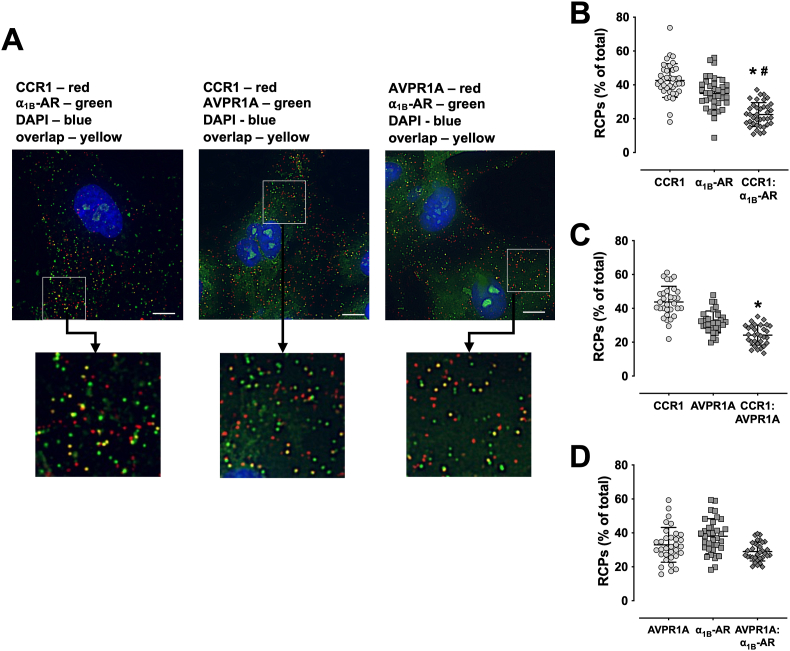
Molecular Boolean analyses of CCR1, α_1B_-AR and AVPR1A in hVSMCs. **A.** MolBoolean staining of hVSMCs for CCR1 and α_1B_-AR (left), CCR1 and AVPR1A (center) or AVPR1A and α_1B_-AR (right). Images show merged red, far-red (pseudo-green) and blue channels, as indicated in the graphs, and are representative of three independent experiments performed on different days. To enhance visibility of RCPs, white squares indicate cell areas that are shown in larger magnification below. Scale bars: 10 μm. **B.-D.** Quantification of RCPs from n = 3 independent experiments per condition, as in A. Horizontal lines indicate mean and SD. RCPs were quantified as % of all (red, green and yellow) RCPs. N = 30–45 vision fields from n = 3 independent experiments were analyzed per condition. **B.** CCR1 (red RCPs), α_1B_-AR (pseudo-green), CCR1:α_1B_-AR: overlapping RCPs (yellow). *: p < 0.05 vs. CCR1. #:p < 0.05 vs. α_1B_-AR. **C.** CCR1 (red RCPs), AVPR1A (pseudo-green), CCR1:AVPR1A: overlapping RCPs (yellow). *: p < 0.05 vs. CCR1. **D.** AVPR1A (red RCPs), α_1B_-AR (pseudo-green), AVPR1A:α_1B_-AR: overlapping RCPs (yellow).

Fig. 3A shows representative merged channel images of the MolBoolean staining of hVSMCs and Fig. 3B–D the quantification of the RCPs from three independent experiments. As compared with our observations in the HEK293T expression system, the percentages of overlapping RCPs for each receptor-receptor combination were lower in hVSMCs. When MolBoolean staining was performed for CCR1 and α_1B_-AR, overlapping RCPs averaged 22.4 ± 7 % of all RCPs (Fig. 3B), which was significantly lower than the percentages of RCPs representing non-interacting receptors. After staining with anti-CCR1 and anti-AVPR1A (Fig. 3C), overlapping RCPs represented 24.2 ± 5.9 % of all RCPs, which is significantly lower than the RCPs for non-interacting CCR1 and undistinguishable from the proportion of non-interacting AVPR1A. MolBoolean staining for AVPR1A and α_1B_-AR showed that overlapping RCPs represented 29 ± 5.5 % of all RCPs (Fig. 3D), which was comparable with the precentages of non-interacting AVPR1A and α_1B_-AR.

## 4 Discussion

In the present study, we utilized the MolBoolean assay to provide initial information on the relative distribution of GPCR heteromers composed of CCR1, α_1B_-AR and AVPR1A in a recombinant system and in hVSMCs. We have validated the selectivity of all primary antibodies for their GPCR targets utilizing established criteria for antibody selectivity previously [12,[14], [15], [16]]. In addition, the quantification of RCPs after labeling HEK293T cells or hVSMCs with mouse or rabbit IgG in the present study suggested that only small proportions of the RCP signals for the receptor targets might be attributable to non-specific signals. Moreover, we showed previously that signals from proximity ligation assays with non-permeabilized hVSMCs are located on the cell surface and do not permit the detection of intracellular proteins [5,7]. Because the MolBoolean assay and PLA are based on the detection of two protein targets with primary antibodies derived in different species, it appears unlikely that meaningful numbers of RCP signals in the present study were located in intracellular compartments.

The estimated distance between two proteins that permits formation of bi-colored RCPs in MolBoolean staining is approximately 40 nm or less [4]. Given that the diameter of a GPCR is approximately 5 nm and GPCR nanoclusters composed of multiple protomers (>10) have been reported [17,18], the detection of overlapping RCPs in MolBoolean assays could represent a heteromeric receptor complex with direct physical interactions between the two receptor partners, the presence of both receptors within hetero-oligomeric receptor complexes composed of up to 8 side-by-side arranged protomers, or no interactions. Thus, further characterization of putative heteromeric receptor complexes represented by overlapping RCPs and identification of heteromer-specific functions are required before such complexes should be assumed [2]. The interpretation of non-overlapping RCPs is less complex, suggests no direct physical interactions between the receptor partners and makes their presence within hetero-oligomeric nanoclusters composed of less than 8 linearly arranged protomers unlikely.

Our findings on recombinant receptors are in good agreement with the previous observation that approximately 80 % of dopamine D2 and adenosine A_2A_ receptors are detectable in overlapping RCPs when the receptors were co-expressed in HEK293T cells [13]. The observation that the percentages of overlapping RCPs were higher in HEK293T cells than in hVSMCs could be explained by overexpression of the receptors on the plasma membrane of HEK293T cells, resulting in a higher density of receptors that are expressed within the distance threshold required to generate a positive signal. Alternatively, cell type specific differences in plasma membrane lipid and cholesterol composition may affect the spatial distribution of the receptor partners and contribute to the observed differences in overlapping RCPs between HEK293T cells and hVSMCs [19].

While the findings on overlapping RCPs in hVSMCs in the present study confirm our previous observations from PLA, the MolBoolean analysis now also suggests that between 30 and 50 % of each receptor partner may be able to participate in the formation of heterodimeric or hetero-oligomeric receptor complexes. This distribution matches well with the observed distribution of chemokine (C-X-C motif) receptor 4 protomers and oligomers in the plasma membrane that has been observed by stimulated emission depletion super-resolution microscopy in naïve CD4^+^ T-lymphocytes [17]. Furthermore, the observation that the percentages of non-overlapping and overlapping RCPs in hVSMCs were very similar for each receptor-receptor combination may further support our previous findings, which indicate that the receptor partners exist in heteromeric complexes composed of all three receptor partners [11].

In conclusion, the MolBoolean analysis of receptor-receptor interactions provides new insights into the spatial distribution of GPCRs in the plasma membrane. We have previously provided several lines of evidence for the formation and functional relevance of heteromeric receptor complexes composed of CCR1, α_1B_-AR and AVPR1A employing various methodological approaches [9,11,12]. Our findings from the MolBoolean analyses of receptor – receptor proximity in the present study expand on our previous observations and now suggest that large proportions of CCR1, α_1B_-AR and AVPR1A may participate in the formation of receptor heteromers. These findings support the concept that such hetero-oligomeric complexes composed of CCR1, α_1B_-AR and AVPR1A are of physiological relevance and provide an explanation for our previous observation that activation of CCR1 differentially regulates G protein signaling and function of agonist stimulated α_1B_-AR and AVPR1A in hVSMCs [12]. Such a phenomenon would not be expected if only small proportions of the receptor partners would form heteromeric receptor complexes.

## Funding

Research reported in this publication was supported by the 10.13039/100000002National Institutes of Health under award number R35GM152056. The content is solely the responsibility of the authors and does not necessarily represent the official views of the National Institutes of Health.

## CRediT authorship contribution statement

**Xianlong Gao:** Data curation, Formal analysis, Investigation, Methodology, Project administration, Validation, Visualization, Writing – review & editing. **Matthias Majetschak:** Conceptualization, Formal analysis, Funding acquisition, Project administration, Supervision, Visualization, Writing – original draft, Writing – review & editing.

## Declaration of competing interest

The authors declare the following financial interests/personal relationships which may be considered as potential competing interests:Matthias Majetschak reports financial support was provided by NIH National Institute of General Medical Sciences. If there are other authors, they declare that they have no known competing financial interests or personal relationships that could have appeared to influence the work reported in this paper.

## Data Availability

Data will be made available on request.
